# Cell signalling regulates dynamics of Nanog distribution in embryonic stem cell populations

**DOI:** 10.1098/rsif.2012.0525

**Published:** 2013-01-06

**Authors:** Yang Luo, Chea Lu Lim, Jennifer Nichols, Alfonso Martinez-Arias, Lorenz Wernisch

**Affiliations:** 1 Biostatistics Unit, Medical Research Council, Cambridge, UK; 2 Department of Genetics, University of Cambridge, Cambridge, UK; 3 Centre for Stem Cell Research, University of Cambridge, Cambridge, UK; 4 Institute of Health Sciences, Universiti Brunei Darussalam, Gadong, Brunei Darussalam

**Keywords:** stochastic system, embryonic stem cell, Nanog regulation

## Abstract

A population of mouse embryonic stem (ES) cells is characterized by a distribution of Nanog, a gene whose expression is associated with the degree of pluripotency. Cells exhibiting high levels of Nanog maintain a state of pluripotency, while those with low levels are more likely to undergo differentiation. Using a cell line with a fluorescence tag for Nanog enables measurements of the distribution of Nanog in an ES cell culture in a stationary state or after a perturbation. In order to model the dynamics of the system, we assume that the distribution of Nanog-GFP for single cells shows distinct attractor steady states of Nanog levels, with individual cells moving between these states stochastically. The addition of synthetic inhibitors of signal transduction induces strong shifts in the distribution of Nanog. In particular, the addition of Chiron and PD03, inhibitors for the ERK and GSK3 signalling pathways, induces a high level of Nanog. In this study, we placed ES cells in different culture conditions, including the above inhibitors, and recorded the change in Nanog-GFP distribution over several days. In order to interpret the measurements of Nanog levels, we propose a new stochastic modelling strategy for the dynamics of the system not requiring detailed knowledge of regulatory or signalling mechanisms, while still capturing the stochastic and the deterministic components of the stochastic dynamical system. Despite its relative simplicity, the model provides an insight into key features of the cell population under various conditions, including the level of noise and occupancy and location of attractor steady states, without the need for strong assumptions about the underlying cellular mechanisms. By applying the model to our experimental data, we infer the existence of three stable steady states for Nanog levels, which are the same in all the different conditions of the cell-culture medium. Noise, on the other hand, and the proportion of cells in each steady state are subject to large shifts. Surprisingly, the isolated effects of PD03 and Chiron on noise and dynamics of the system are quite different from their combined effect. Our results show that signalling determines the occupancy of each state, with a particular role for GSK3 in the regulation of the noise across the population.

## 1. Introduction

The development of an organism relies on the coordination of transitions of cells through several states that lead to the final differentiated cell types, which make up tissues and organs. Over the last few years, our understanding of this process has improved with our increased ability to gather information about the changes of gene expression that are associated with particular states [1–4]. The role of noise (spontaneous fluctuation in the levels of transcription of particular genes) in triggering transitions has been emphasized in recent studies [5–8]. In these studies, noise is represented as a stochastic term in a stochastic dynamical system driven by the activity of gene regulatory networks and influenced by signalling pathways.

**Figure 1.**
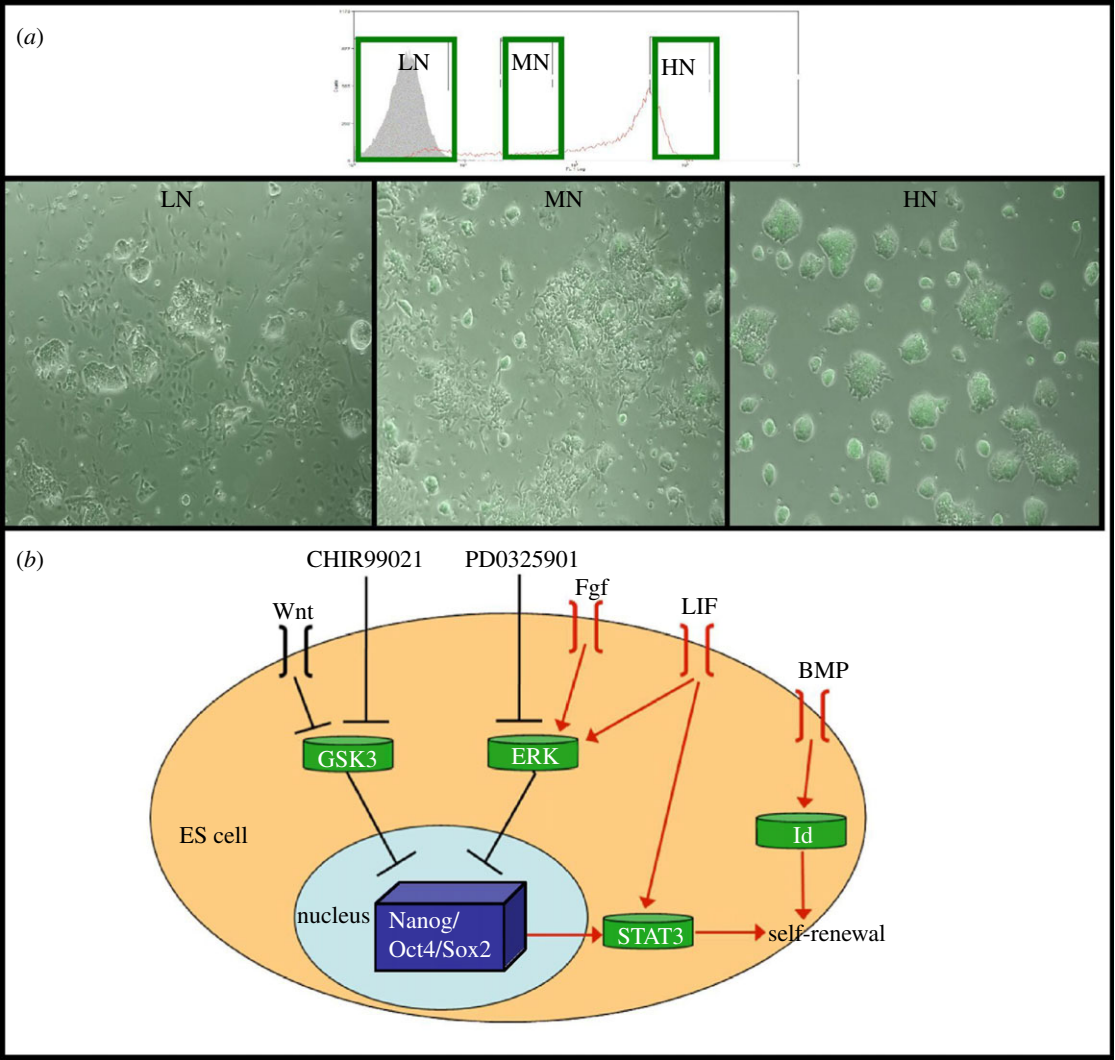
Morphology of targeted Nanog-GFP, clone A (TNGA) cells with different levels of Nanog-GFP and key signalling pathways required for maintaining pluripotency. (*a*) TNGA cells with three different levels of Nanog-GFP expression were sorted from a steady-state population (red lines) and cultured for 3 days. As the level of Nanog-GFP increases from low Nanog (LN) to high Nanog (HN) (left to right), the proportion of differentiated cells has visibly reduced as seen from the absence of flat differentiated cells from the HN cells. MN, middle Nanog. (*b*) A schematic of the signalling pathways shown to be important in maintaining pluripotency in mouse ES cells. The LIF-Stat3 and BMP4-Id pathways together maintain self-renewal and are thought to function by inhibiting mesodermal and neural differentiation, respectively [19]. Pluripotency can also be maintained by the use of two inhibitors, PD0325901 and CHIR09921 [20]. PD03 inhibits the Fgf-MEK pathway, which is vital for ES cells to commit to differentiation [21]. CHIR, on the other hand, targets GSK3b and, by doing so, stimulates the Wnt pathway [22] as well as possibly promoting cell metabolism [20].

Mouse embryonic stem (ES) cells, which are cultured cell populations derived from the blastocyst, are pluripotent, that is, they can develop into any cell type of the embryo and can propagate this ability [9]. Experiments with ES cells have provided examples of the dynamics of cell states during cell-fate decisions [10–16]. The expression of the transcription factor Nanog is often used as a marker for pluripotency. Nanog exhibits high variability from cell to cell; high levels of Nanog are associated with pluripotency, while low levels are associated with a tendency to differentiate [10,13,17]. Cells change their levels of Nanog over time but an ES cell population tends to equilibrate towards a distribution that remains constant under standard culture conditions ([10,13,18]; figure 1). This distribution is very sensitive to signals provided by the culture medium. In particular, growth in the presence of two inhibitors, PD03 and Chiron, dramatically reduces the heterogeneities and enriches the population in cells with high levels of Nanog [17,23].

The biochemical activities of these molecules are well known. PD03 inhibits signalling by FGF, which has been shown to promote commitment to differentiation of ES cells, and inhibition of GSK3 increases the activity of β-catenin and potentiates cell viability (see figure 1 for an overview) [20–22]. Work from Smith and co-workers [20] has shown that addition of these two synthetic inhibitors of signal transduction to N2B27 maintains cells in a highly pluripotent state. However, what the effects of these inhibitors are on the states associated with Nanog expression and, more importantly, on the dynamics of the system has not been investigated.

It is known that, under normal self-renewing conditions, cells from any part of the distribution will reform the original distribution over time and it is thought that this behaviour is associated with the regulative properties of an ES cell culture [10,13]. In order to analyse the effects of these inhibitors on Nanog distribution and its regulative properties, we obtained time-series fluorescence-activated cell sorting (FACS) measurements of the distributions of Nanog expression levels for ES cells placed in different media conditions. We used stationary populations but, more significantly, we selected specific subpopulations and monitored their evolution under different culture conditions.

### 1.1. Modelling strategy

Models of the dynamics of ES cell populations can potentially provide insights into the function of and interactions between the elements of the signal transduction pathways and gene regulatory networks that define pluripotency. A widely used modelling strategy is to postulate a mechanistic model based on pathways or networks with precise kinetics that leads to dynamical models in terms of deterministic or stochastic differential equations derived from regulatory relationships [10,24,25]. However, these models rely on detailed knowledge of the architecture of the network and on mathematical descriptions of regulatory interactions, which are often difficult to justify directly in terms of experimental data. Moreover, such models are highly parametrized and flexible, and consequently very different models can explain the data at hand equally well, complicating the choice of the correct model. Such choice has to rely on additional assumptions and knowledge outside the data. Consequently, it often remains doubtful whether anything new has been learned from the data that have not already been incorporated in the model *a priori*. Extrapolating from highly parametrized models is problematic as well, as overfitting will diminish their predictive power. On the other hand, reduction of the number of parameters is only possible after making even stronger theoretical assumptions about the details of the regulatory model.

In this study, we propose an alternative modelling strategy. Our aim is to capture the essence of a stochastic dynamic process with a minimum of model assumptions. One of our key modelling assumptions is that the distribution of Nanog-GFP observed in experiments is the result of stochastic fluctuations. A stochastic dynamical system has two major components: the deterministic part, represented by a drift term; and the stochastic part, represented by a noise term. Instead of modelling the drift term through differential equations derived from known or hypothesized pathways or networks, we assume a more general functional form. For systems with one variable, such as the Nanog expression data from our experiments, the drift term can be represented as the derivative (slope) of a *potential function*, if we assume that a stationary distribution exists.

**Figure 2.**
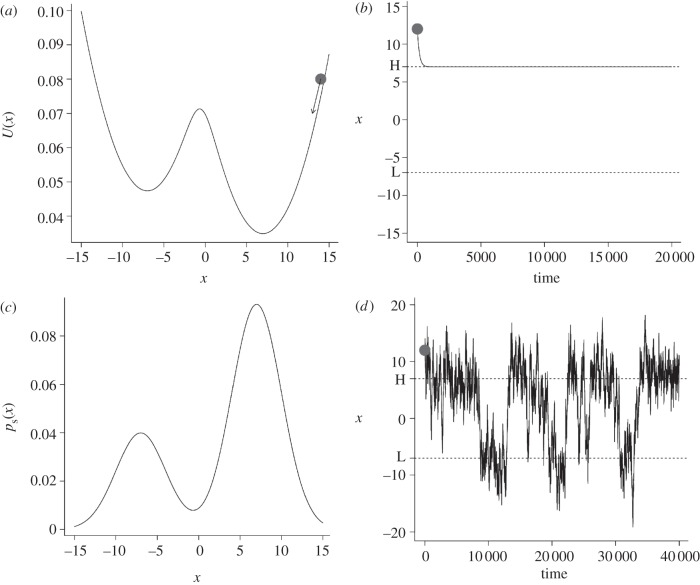
The effect of a potential on the dynamics of a system. (*a*) A system (symbolized by a ball) is driven by an energy potential towards the nearest local minimum (in a mechanical model, the movement of a mass under gravity in a highly viscous fluid that dissipates the kinetic energy). The speed or drift is proportional to the inclination or gradient of the potential function. (*b*) Without noise, the system converges to the local minimum. (*c,d*) With noise, the system can overcome the energy barrier and moves between the local minima and intermediate states, sampling from each state according to a distribution determined by the potential and the noise. The local minima in the potential correspond to local maxima in the state distribution.

Figure 2 illustrates these concepts: a potential function has two stable steady states (local minima). The system, a ball in the figure, moves deterministically towards the closest steady state, the closest local minimum in the potential function, under the influence of the inclination (gradient) of the potential (figure 2*b*). In a physical interpretation of this process, the ball would move under gravity downhill in a highly viscous fluid that dissipates the kinetic energy. Unless the underlying potential function changes through outside influence, the system would stay in this steady state in a deterministic model. However, in a stochastic model, noise gives the ball random jolts and can drive it off the stable steady state. Higher levels of noise allow the ball to overcome the barriers between stable states and lead to rapid fluctuations between the stable states (figure 2*d*), giving rise to a distribution that represents the time average of the behaviour of the system (figure 2*c*). The larger the noise, the flatter this distribution becomes, even though the underlying potential remains the same. Thus, the system has two significant components: the potential and the noise, and its behaviour is a result of the interplay between both.

Our approach represents an arbitrary stationary distribution non-parametrically by a mixture of Gaussian distributions, as in figure 2*c*, and a potential similar to the one in figure 2*a* can be derived from this representation (given a certain noise level). We thus avoid any need to develop a detailed kinetic regulation model but still obtain a representation of the deterministic dynamics. In order to make such a model identifiable from the data, we have to make a few technical assumptions. First, the noise term is independent of the Nanog level. Because we fit our model using logarithmic Nanog levels, we therefore implicitly assume that noise intensity is proportional to the level of Nanog expression. However, alternative functional dependencies of the noise intensity on Nanog levels can be easily incorporated into our framework if that seems desirable. A more serious restriction of our approach is that it applies to systems with one variable only: the existence of a potential is not guaranteed for higher dimensional systems, even if a stationary distribution exists. However, even in such cases, the assumption of the existence of a potential is likely to result in a good approximation (for example, in three dimensions an approximation based on a Helmholtz decomposition could be considered).

**Figure 3.**
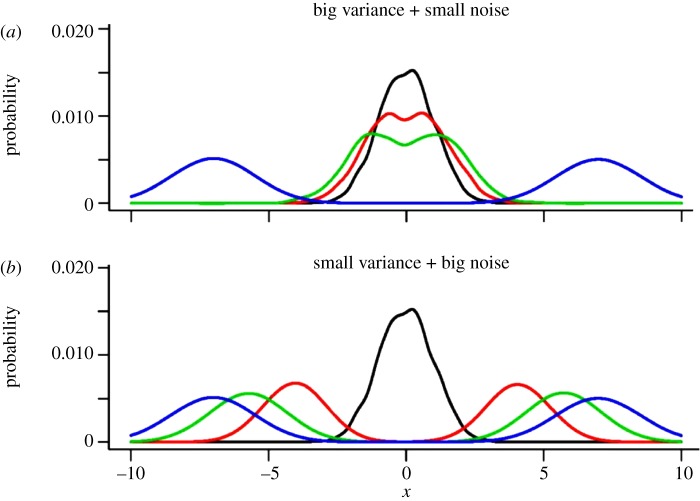
The effect of noise on the speed of time evolution. Shown are kernel density estimates for simulations for two different stochastic systems. (*a*) The minima in the potential function are wide but the noise is small. (*b*) The minima are narrow but the noise is large. In the limit, both systems have the same stationary distribution (blue), but the speed of evolution is quite different. This phenomenon enables the identification of potential and noise parameters from time-series data. Black line, day 0; red line, day 1; green line, day 2.

Stationary data are not sufficient for the identification of the deterministic and stochastic components in our model. Intuitively, an observed stationary distribution can be either the result of a deep potential function but large noise or a flat potential function and small noise. On the other hand, time-course data allow us to distinguish between these possibilities and to identify both, the deterministic and the stochastic components, as long as there is enough change in the population distribution over the time course. Figure 3 illustrates this by showing the outcome of stochastic simulations with two different noise levels and two different potentials that are chosen so that the stationary distribution is the same. The rate of time evolution is very different, and, as the figure illustrates, identification of noise and potential is feasible from dynamic but not from stationary data.

An approach using a flexible function for the drift term is, of course, not suitable for inference on the details of the underlying regulatory or signalling mechanism. Moreover, we use only a one-dimensional model with a comparatively simple representation of the stationary distribution as a mixture of Gaussians. However, even such a straightforward model allows us to extract surprising and important features of signalling in the Nanog system, not at all obvious from looking at the raw data, as shown in §§3 and 4. Despite its simplicity, it also illustrates the main feature of our modelling strategy: to extract as much as possible about the *structure* of a system, and not only parameters, from data.

For model comparison and estimation of parameters, we use a statistical technique, nested sampling [26], that has gained recent interest in some areas such as cosmology [27]. The advantages of this Bayesian method are that it is very efficient computationally (much more so than Monte Carlo methods) and that it provides a model likelihood along with posterior parameter estimates (unlike Monte Carlo methods).

We apply the model to time-course data of Nanog-GFP in order to reconstruct the effect of the two inhibitors and their combination on the location and depth of the attractor states and on the level of stochastic noise. Despite some statistical technicalities, our modelling approach is reasonably simple and straightforward to apply: estimating parameters, for example, turns out to be quite robust, and, owing to its generality, the method can be easily adapted to other systems. Software implemented in the R statistical language is available from the authors.

## 2. Material and methods

### 2.1. Stochastic model

The *Fokker–Planck equation* (FPE) describes a continuous Markov process by providing the time evolution of the probability density function for the system [28]2.1
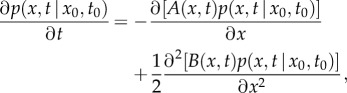
where 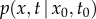 represents the probability density function for state *x* (Nanog levels in our example) at time *t*, when the system is started in *x*_0_ at time *t*_0_, *A*(*x,t*) is the *drift* and *B*(*x,t*) is the *diffusion* coefficient. The stationary distribution 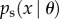, defined by parameters *θ*, is characterized by a zero *probability current*2.2
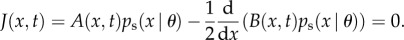
Intuitively, once a collection of cells has reached a stationary distribution, at each (Nanog) level, the number of particles (cells) moving to the left (reducing Nanog level) equals the number moving to the right (increasing Nanog level); so the overall number of cells with a certain Nanog level stays the same.

We model the stationary distribution from equation (2.2) non-parametrically as a mixture of *k* Gaussian distributions,2.3
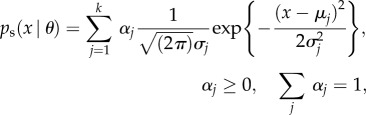
with parameters 
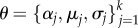
. For identifiability, we assume a time- and position-independent diffusion coefficient *B*(*x,t*) = 2*B*. From equation (2.2), for zero probability current *J*(*x,t*) = 0, the drift term can be expressed as2.4
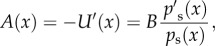
where *U*(*x*) is the *potential function* *U*(*x*) = −*B* log*p*_s_(*x*). The stationary distribution *p*_*s*_(*x*) is determined by the potential *U*(*x*) and noise intensity *B*. Conversely, from the stationary distribution *p*_s_(*x*) alone, *U*(*x*) and *B* cannot be uniquely derived as, from equation (2.4), a factor for the drift can compensate changes in *B*.

### 2.2. Inference of parameters

At each time point *t*_*r*_, *r* = 0, … , *T* we have a set *x*_*r*_ = *x*_1,*r*_, … , *x*_*n_r_*__*,r*_ of *n*_*r*_ measurements (here Nanog-GFP levels in single cells with *T* = 3). A set of parameters 
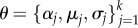
 defines a distribution 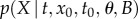 of levels via equations (2.1), (2.3) and (2.4). The likelihood function is2.5
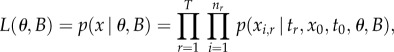
where 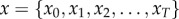. The dataset *x*_0_ is used for a kernel density estimate of the initial distribution at *t*_0_. In our case, the samples at different time points are independent of each other, which justifies the multiplication of probabilities at different time points. The measurements are undertaken on fresh samples from the total population of cells at each time. Samples at different time points are therefore independent of each other. However, all sample distributions depend on the initial sample distribution *x*_0_ at time *t*_0_, which is under experimental control via FACS sorting.

There are several ways to obtain 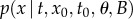 numerically; for example, by solving the second-order partial differential equation (2.1) [28]. Here we sample stochastic trajectories from the stochastic differential equation corresponding to the FPE [28,29] for 1000 initial starting points drawn randomly from *x*_0_, and fit a kernel density estimate to the results at time point *t* to approximate 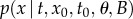. This scheme turned out to be very robust in that different runs converge on the same parameter estimates.

For the inference of the model parameters via equation (2.5) and model comparison, we used a nested sampling procedure [26,27], which turned out to be much more efficient than alternative inference methods such as Markov chain Monte Carlo methods (for details see the electronic supplementary material notes).

### 2.3. Embryonic stem cell culture

E14IVc and targeted Nanog GFP, clone A (TNGA) ES cells were a kind gift from Austin Smith's laboratory and were described previously [13]. TNGA cells contain a GFP reporter that is fused to a puromycin gene, which is inserted into the Nanog locus, with the one Nanog gene left intact.

All ES cells were maintained in Glasgow minimal essential medium (Sigma) supplemented with 10 per cent fetal bovine serum (Sigma), 1 × MEM non-essential amino acids (Invitrogen), 100 µM 2-mercaptoethanol (Sigma) and 5 × 105 units ml^−1^ ESGRO (Millipore) on gelatinized tissue-culture flasks. For TNGA cells, additional treatment with puromycin (1 µg ml^−1^) for three consecutive passages led to the removal of any differentiated or wild-type cells that had lost their transgene.

ES cells were then cultured in serum-free conditions (N2B27 supplemented by LIF and 10 ng ml^−1^ BMP4 (R&D)) [19] for two or three subsequent passages before they were sorted or used in an experiment.

### 2.4. Flow cytometry sorting

Cells were prepared for flow cytometry sorting using a Beckman Coulter high-speed cell sorter by harvesting the cells using Accutase (Invitrogen) and diluting in Dulbecco's minimal essential medium-F12 (Invitrogen) supplemented with 0.2 per cent bovine serum albumin and 100 µM 2-mercaptoethanol. Cells were then resuspended in LIF and BMP4, and filtered using a 30 µm mesh. Live single cells were selected for further analysis on the basis of FSC/SSC/pulse width characteristics after prior confirmation with a live/dead dye, Topro3 (Invitrogen), that these characteristics yielded live single cells.

The cell sorter was calibrated each time by using fluorescence beads. Parental E14IVc ES cells were used as a negative control because they display a level of autofluorescence; this was used to calibrate the laser intensities so that they were within the range of 10^0^–10^1^. Cells with fluorescence levels within this range were considered to be negative (i.e. low Nanog; LN). Levels between 10^2^ and 10^3^ were considered high (high Nanog; HN). Middle Nanog (MN) cells have an intermediate level of Nanog-GFP, lying between 10^1^ and 10^2^. The sorted cells were re-analysed to check the purity of sorting (which is above 98%).

### 2.5. Flow cytometry analysis of time course

**Figure 4.**
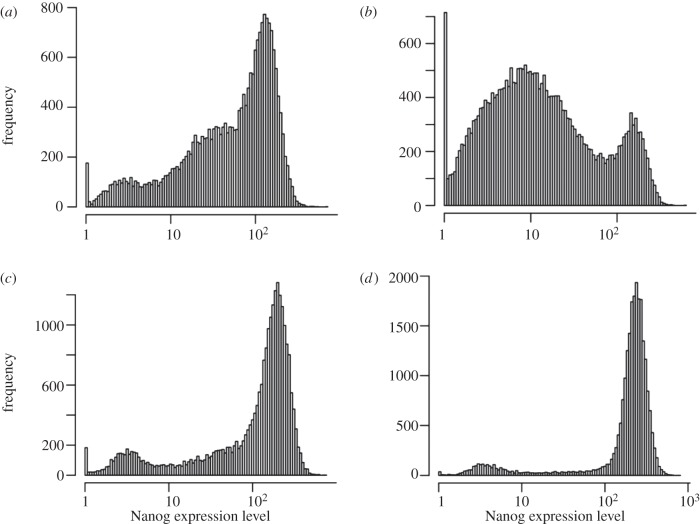
Nanog expression distribution of unsorted ES cells. Unsorted cells were plated from (*a*) LIF+BMP4 into media containing either (*b*) PD03, (*c*) Chiron or (*d*) 2i, or LIF+BMP4. The figure shows the profiles of TNGA cells after 4 days for each culture condition.

**Figure 5.**
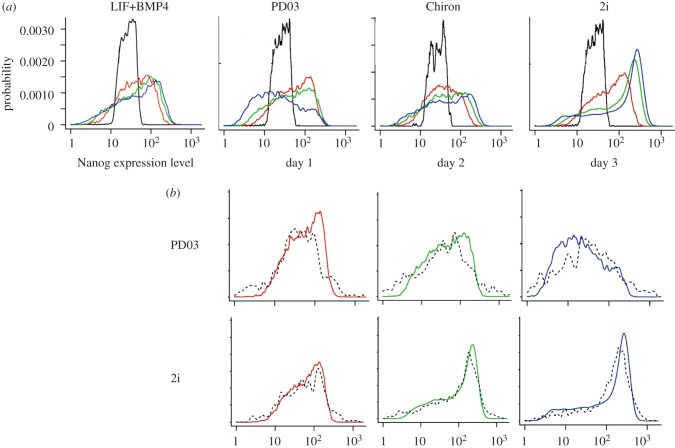
Nanog expression distribution in the middle Nanog (MN) fraction of sorted ES cells. Sorted cells from the MN fraction were plated from LIF+BMP4 into media containing either PD03, Chiron or 2i, or LIF+BMP4 (for details, see §2). (*a*) Experimental data (days 0–3) of ES cells cultured in LIF+BMP4, PD03, Chiron and 2i. (*b*) Distributions of observed Nanog expression levels (solid lines) and predicted levels (dashed lines) sampled from the fitted theoretical model for PD03 and 2i. Black line, day 0; red line, day 1; green line, day 2; blue line, day 3.

Cells were plated into N2B27 supplemented with either an MEK inhibitor (termed PD03 in the following), 1 µM PD0325901 (Division of Signal Transduction, University of Dundee, UK), GSK3 inhibitor (termed Chiron in the following), 3 µM CHIR99021 (Division of Signal Transduction, University of Dundee), 2i (a combination of 1 µM PD0325901 and 3 µM CHIR99021) [20] or LIF and BMP4 (LIF+BMP4). Over a subsequent period of 3 days, the media were changed daily and cells were prepared for analysis in a manner similar to that of cell sorting, using a Beckman Coulter CyAN ADP analyser. The plating in different conditions was done twice, once with an unsorted population of cells (figure 4) and once with the MN fraction after sorting (figure 5*a*).

The cell analyser was calibrated for each day as well. For day 0 (day of sorting), sorted cells that were not plated for the time course were analysed to give a starting profile, together with beads of fixed fluorescence (BD Sphero rainbow beads). For days 1–3, the median of the beads on day 0 was then used to set up and standardize the PMT voltages of GFP fluorescence before any readings were taken, in order to compensate for any daily variation of the laser intensity; 10 000 cells were analysed each time.

## 3. Results

### 3.1. Structure and dynamics of a pluripotent cell population

Profiling of Nanog-GFP by FACS indicates that, in optimal conditions (for example, minimal N2B27 medium with LIF and BMP4 (LIF + BMP4)), a pluripotent culture contains two discrete populations of cells: some with high levels of Nanog (HN)—which favour a pluripotent state—and some with low levels of Nanog (LN)—which is prone to differentiation [10,13]. When LIF and BMP4 are removed from the culture, ES cells drift towards differentiation and this is reflected in a quick shift of the profile of Nanog-GFP expression towards the LN peak: after 2 days in N2B27, more than 80 per cent of the population occupies this position. This observation establishes a basis to test the influence of different signalling systems on pluripotency [10].

When TNGA cells were switched from LIF+BMP4 to different culture conditions, the profile of Nanog-GFP changed. Figure 4 shows the profiles of TNGA cells in the four different culture conditions (LIF+BMP4, Chiron, PD03 and 2i) after 4 days. Beyond the fourth day, the distributions change little (except perhaps for PD03) and can be assumed to approach a stationary state soon thereafter. In 2i, the profile of TNGA cells is very skewed towards the HN population with an almost non-existing LN population, which is characteristic of LIF+BMP4. Chiron maintains the cells in the LIF+BMP4 state with some changes in the HN peak and the decrease of a prominent hump that lies between the two peaks. On the other hand, PD03 alone exhibits a very different profile: rather than a maintenance of the LIF+BMP4 profile, as might have been expected from the reported increased pluripotency of the culture [20,21], we observe an increase in the populations with lower levels of Nanog; in this condition, a third clear MN population can be observed between the HN and LN, which is present but very reduced in other conditions.

Our observations suggest that the description of the TNGA culture as a mixture of two populations might be an oversimplification. To establish quantitatively how many subpopulations exist in a self-renewing culture, we assumed that the stationary distribution of Nanog-GFP is a mixture of Gaussian distributions and calculated the Bayes factor for Gaussian mixtures of two, three and four components, fitted to the profiles of TNGA cells shown in figure 4. In the cases of LIF + BMP4, Chiron and PD03, the analysis favours a mixture of three components over two or four (table 1). The distribution resulting from the culture in 2i requires a simpler distribution with two Gaussian components, indicating that this set of conditions affects drastically one of the populations. Overall, we conclude that a mixture of three Gaussian distributions is the most suitable model among the three competing models to describe the Nanog-GFP profiles for all but possibly the 2i condition. However, there is little harm in assuming more components than necessary, because the importance (occupancy) of an unnecessary component will just be estimated as small.

**Table 1.** Model comparison for the number of mixtures component. The table shows the Bayes factors for the number of Gaussian components needed to model the distribution of Nanog-GFP data in various culture conditions: *B*_32_ is the odds of three components over two, *B*_34_ the odds of three components over four and *B*_42_ the odds of four components over two. Odds between 1 and 3 are marginal, between 3 and 10 substantial and above 10 strong.

	LIF+BMP	Chiron	PD03	2i
*B*_32_	113	713	39.2	1/38560
*B*_34_	2.03	2.33	2.14	1/6247
*B*_42_	55.7	306	18.3	1/6.17

Having established the number of components in the population, we are in a position to probe for the shape of the potential and the intensity of the noise associated with the system, and to study the effect that the different signalling systems have on these parameters. However, as discussed earlier, the potential cannot be identified independently of the noise component, unless there is enough change in the distribution over the time in which samples are collected. A number of experiments show that the HN population is very stable, whereas the LN population differentiates and exhibits a low number of cells returning to the HN population under self-renewal conditions [10]. By contrast, the MN population exhibits rapid changes under all conditions towards both HN and LN (see below). For this reason, we decided to focus our study on the dynamics of the MN population which should provide the necessary information for estimating coefficients: mean and standard deviation of each component and their occupancies defining the deterministic potential, as well as a stochastic noise term.

### 3.2. Estimation of potential function and noise

A stationary culture of TNGA cells grown in LIF and BMP4 was FACS sorted to obtain a population enriched in MN. These cells were then placed in different culture conditions (LIF and BMP4, PD03, Chiron, and 2i) and the developing distributions of Nanog-GFP were measured on three consecutive days. As an illustration, figure 5 shows kernel density estimates for the raw data (solid line) and sampled data from the fitted models (dashed lines). A first impression of the changes can be obtained by visual inspection of the plots in figure 5. The HN population is dramatically reduced in PD03, and becomes distributed over MN and LN in about equal amounts. This agrees with independent experimental findings that with PD03 alone ES cells progress to a state of more homogeneous Nanog expression [21]. Chiron seems to induce a clear rapid shift of about half of the MN population into the HN state, while 2i moves almost all cells to the HN state.

In order to obtain a more precise insight into the nature of the changes, we have to inspect the model parameters inferred from the data under each of the four conditions, as shown in table 2. The relevant parameters are: occupancy *α*, mean *μ* and variance *σ*^2^ of each Gaussian component and the noise intensity *B* of the system. A summary of the results for the stochastic model under the four different conditions is listed in table 2 (the 95% credible intervals of the estimated parameters are shown in the electronic supplementary material, table S2).

**Table 2.** Three component models for different culture conditions. Parameters *α*, *μ* and *σ* are the percentage, mean and variance of the Gaussian components of the mixture density for low, middle and high Nanog expression level—LN, MN, HN, respectively. 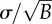 provides an indication of the width of the potential.

	*α*_LN_ (%)	*α*_MN_ (%)	*α*_HN_ (%)	*μ*_LN_	*μ*_MN_	*μ*_HN_	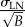	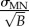	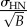	*B*
LIF + BMP	12.7	43.1	44.2	0.807	1.80	2.22	0.955	0.673	0.571	0.230
PD03	43.9	46.5	10.6	0.524	1.71	2.38	0.187	0.393	0.199	0.860
Chiron	10.3	27.5	62.2	0.601	1.65	2.21	0.831	1.05	0.246	0.186
2i	2.68	5.67	91.6	0.598	1.80	2.32	0.195	0.818	0.382	0.303

Occupancies *α*_LN_, *α*_MN_, *α*_HN_ represent the percentages of cells that occupy the LN, MN and HN valleys in the stationary distribution. With reference to figure 2, an occupancy close to 1 indicates a deep value in the potential function and the existence of a strong attractor. *B* represents the amount of stochastic noise that enables these transitions. The scaled variance *σ*^2^/*B* measures the width of the valleys in the potential function, while *σ*^2^ is the width of the mixture component in the stationary distribution (including variance owing to noise). A larger noise will result in a wider Gaussian distribution, and vice versa.

We are interested in the effect of the inhibitors on these parameters. Table 3 lists the posterior probabilities for the differences seen in table 2, considering the uncertainty in the estimation of parameters. The numbers in bold indicate probabilities of less than 0.1 or greater than 0.9. That is, the corresponding parameter values are likely to be substantially different for the respective culture conditions.

**Table 3.** Comparison of parameters across culture conditions. The probability *P*(*θ*_*A*_ < *θ*_*B*_) that the parameter *θ* labelling the column is smaller in condition *A* than condition *B* for conditions *A* and *B* labelling the row. Probabilities above 0.9 or below 0.1 are significant and are highlighted in bold.

	*α*_LN_	*α*_MN_	*α*_HN_	*μ*_LN_	*μ*_MN_	*μ*_HN_	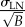	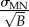	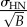	*B*
LB < Chiron	0.371	0.331	0.677	0.276	0.190	0.532	0.523	0.836	0.538	0.229
LB < PD03	**0.999**	0.604	**0.010**	0.152	0.143	0.847	**0.016**	0.124	0.122	**0.994**
LB < 2i	0.302	**0.052**	**0.939**	0.161	0.479	0.831	**0.015**	0.676	0.248	0.715
PD03 < 2i	**0.004**	**0.021**	**1.000**	0.552	0.847	0.299	0.433	**0.967**	0.769	**0.011**
PD03 < Chiron	**0**	0.396	**0.990**	0.848	0.856	0.153	**0.963**	**0.986**	**0.949**	**0.006**
2i < Chiron	0.618	**0.938**	0.175	0.132	0.200	0.217	0.459	0.765	**0.927**	**0.074**

A striking observation is that the positions of the three mixture centres *μ*_LN_, *μ*_MN_ and *μ*_HN_ as the centres of the MN, LN and HN populations are very similar in all conditions. Any differences are not statistically significant (table 2). Considering the very different dynamic evolutions of the MN population in the four conditions, the similarity of the location of the three mixture centres lends support to the hypothesis that there are three distinct steady states that are central to the Nanog system and are independent from the culture conditions and not susceptible to regulation by signal transduction.

However, the different conditions have effects on the scaled variance *σ*^2^/*B*, the width of each state. Overall, Chiron is widening the LN and MN states in particular compared with the narrowing effect of PD03. Both are narrowing the HN state. Finally, PD03 drastically increases *B*, the stochastic noise, while Chiron reduces it slightly, although not significantly compared with LB+BMP4. The noise in 2i, which is a combination of PD03 and Chiron, lies in between that of PD03 and Chiron. The increase in noise from Chiron to 2i with the addition of PD03 is significant. Overall, it seems the noise level of 2i is the result of competing pulls in different directions by Chiron and PD03.

**Figure 6.**
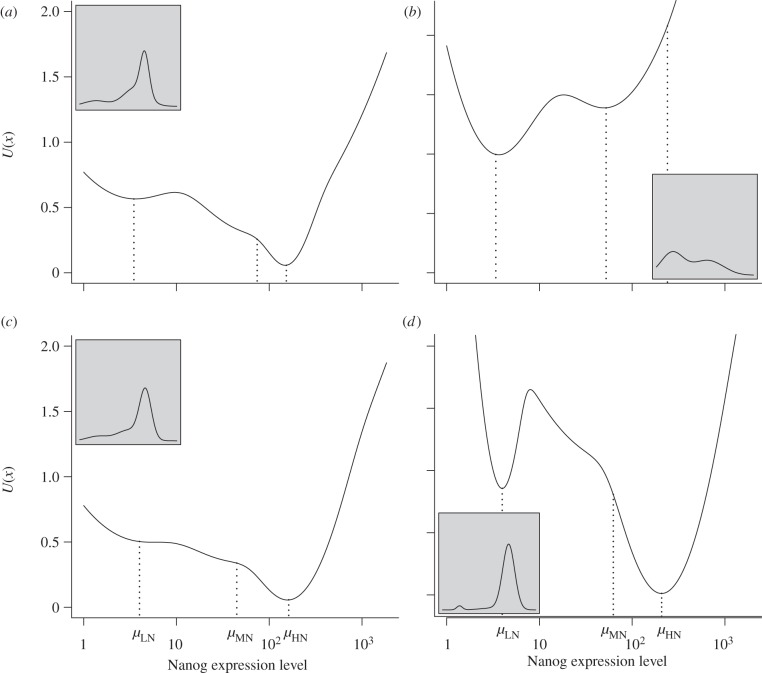
Predicted stationary distributions of Nanog expression levels in different culture conditions. The figure shows the potential landscape of Nanog expression level in different culture conditions using the fitted models (table 2). The distribution associated with each potential and noise is shown in the smaller panel for each figure. The positions of the inferred centres of the three mixture components for each condition are indicated as *μ*_LN_, *μ*_MN_ and *μ*_HN_. (*a*) LIF+BMP4; (*b*) PD03; (*c*) Chiron; (*d*) 2i.

Potential functions for each condition are shown in figure 6. In 2i, most of the TNGA cells exhibit a HN expression level, which is reflected in a deep HN valley. By contrast, PD03 has a rather shallower HN valley. The insets show the corresponding stationary distributions. Interestingly, while PD03 shows a pronounced LN attractor steady state (and an MN steady state) and Chiron a pronounced HN steady state, their combination in 2i shows features of both, steady states at LN and HN. Figure 6 also shows that Gaussian mixture components not necessarily are local minima in the potential function or attractor steady states. However, all three components (LN, MN and HN) show very pronounced local minima in at least one of the conditions. Most notably is a clear attractor steady state at the MN point for PD03.

The models can be used to make predictions to some degree. The predicted stationary distributions as inferred from the time-series data starting with a narrow MN distribution (figure 5) can be compared with the stationary distributions obtained from non-gated whole population cultures under the various inhibitor conditions (figure 4). The predicted distributions capture the general outline of whole-population stationary distributions reasonably well: table 4 lists the log-likelihoods when the predicted stationary distribution for each of the four media (rows) is used to calculate the probability of the four experimental datasets (in the columns). We would expect that the estimated model for each medium predicts the experimental data from the same medium best—that is, the highest likelihood in each row appears along the diagonal of the table, which is indeed the case (highlighted numbers in table 4).

**Table 4.** Predicted log-likelihood of stationary experimental data under dynamical models. The rows contain measurements from a stationary distribution under the corresponding culture conditions (figure 4). The columns contain models obtained from independent dynamic experiments under culture conditions (figure 5). Each entry shows the log-likelihood of the model in the column applied to the data in the row. The highest likelihood for each dataset, the maximum in each row (highlighted in bold), should be achieved by a model trained on separate data under the same condition.

	LIF+BMP4	PD03	Chiron	2i
LIF + BMP4	−**30 655**	−39 091	−31 680	−45 5367
PD03	−59 980	−**43 988**	−62 506	−111 399
Chiron	−32 127	−48 926	**−27 572**	−35 164
2i	−30 494	−53 451	−24 947	−**17 012**

**Figure 7.**
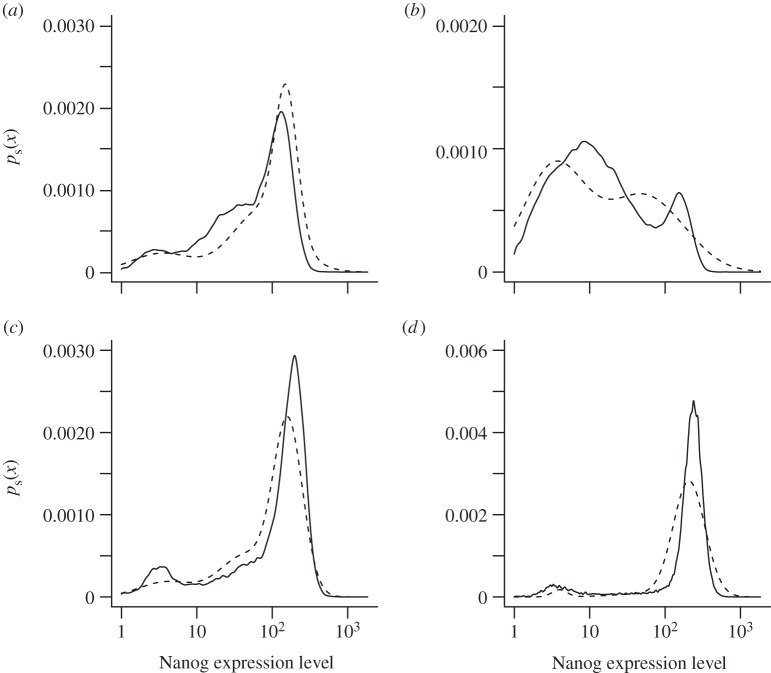
Fitted stationary distributions from MN sorted populations against experimental distributions from unsorted populations. Shown are the predicted stationary distributions (dashed) using the models for the dynamics of the MN fractions (cf. figure 5) against the experimentally obtained stationary distributions (solid) of unsorted whole populations (cf. figure 4) in the same four conditions. (*a*) LIF+BMP4; (*b*) PD03; (*c*) Chiron; (*d*) 2i.

## 4. Discussion

Recent discussions of mechanisms of cell-fate decisions in general and in stem cell populations have made use of the concept of the *epigenetic landscape*, as first proposed by Waddington [30], where the differentiation process of a cell is described by the trajectory of a ball rolling down a potential landscape with valleys and hills representing local minima (steady states, attractor states) and local maxima (energy barriers, as in figure 2) that change with time [31]. However, with rare exceptions in which paths of differentiation have been experimentally inferred [5,32], the use of this concept is largely metaphorical. Our modelling approach enables the inference of the shape of the potential function directly from data as well as the noise level without the need to know the exact kinetic relationships of the components of the underlying system. We applied this approach to measurements of the expression of a GFP reporter for Nanog, a key regulator of pluripotency (figure 6).

The shape of the derived potential suggests that an ES cell population can be described by a mixture of three different subpopulations of a stochastic dynamical system that represent three attractor steady states, each defined by specific levels of Nanog. The choice of Gaussian distributions is for simplicity and convenience as well as to capture the idea of subpopulations in the stationary distribution. Note that a mixture component does not necessarily correspond to a local minimum in the potential function. Alternative choices to Gaussians for mixture components are possible. For example, components that try to model local maxima in the stationary distribution as distinct components might be able to capture distinct attractor steady states in the potential more accurately. However, we feel justified in talking about a distinct attractor steady state for MN because the PD03 condition clearly shows a steady state in the potential at MN (figure 6). An earlier model had considered only two attractors [10]; however, model comparisons with different numbers of components suggest that the introduction of an MN population is a better fit to the data. This MN population can be observed experimentally, shown to have the properties of an attractor, albeit a weak one, and can be shown to play a crucial role as an intermediate state in the transition between HN and LN in our model.

The analysis provides some insight into the effect of signalling on the dynamics of the population. It has been shown that simultaneous inhibition of GSK3 and MEK (2i conditions) results in a very homogeneous population with a high degree of pluripotency [20] associated with a dramatic reduction of the LN population, an elimination of the MN and an accumulation of cells at HN [23]. The potential function under this condition reveals that 2i promotes an increase in the depth of the HN valley, which would make it more difficult for the cells to leave this state. Surprisingly, each of the components of 2i exert an opposite effect on the landscape and the noise.

The potential function for Chiron is very similar to that for LIF and BMP4 with slightly reduced noise and increased occupancy of HN. On the other hand, ERK inhibition by PD03 leads to a very flat landscape owing to high noise (the *B* value of 0.86 for PD03 is significantly higher than that of 0.23 for LIF+BMP4; table 4). Under the PD03 condition, there is comparatively rapid stochastic exchange of cells between all three steady states. The characteristics of the system in 2i suggest that Chiron exerts the dominant effect in the combination. Furthermore, as the effects of these signalling pathways are thought to reflect the activity of the cells under normal conditions [20,22], the lack of additivity of the effects that we have described indicates that there may be cross-regulatory interactions between ERK and GSK3 that are involved in the maintenance of self-renewal. It will of interest to pursue this at the molecular level.

Chiron works through inhibiting GSK3 and, in the context of ES cells, the major target of this inhibition is the activity of β-catenin [22]. Interestingly, it has been suggested that Wnt/β-catenin signalling plays an important role in the regulation of heterogeneities and noise during cell-fate transitions [1]. One of the major effects of Chiron is to keep cells in the HN state with a very low degree of heterogeneity as reflected in the high occupancy *α*_HN_ and low noise *B* providing some support for the notion of Wnt/β-catenin signalling as a controller of noise.

In summary, we propose a new modelling approach to stochastic dynamical systems that relies on as few modelling assumptions as possible while still enabling inference of interesting properties of the system. We only assume the existence of noise and a potential function of an arbitrary shape. Applying this approach to time-series data of Nanog-GFP expression under various conditions showed that most likely there exist three steady states that stay the same under all conditions. What changes is the proportion of cells around each of these states and the noise level. We were able to predict the stationary distributions from the time-series data reasonably accurately. Predictions about transition times of single cells from one steady state to the next, which can also be inferred from our models, will be tested in a future project.
